# COVID-19 knowledge, attitude, and practice in combating TB and COVID-19 in Cameroon

**DOI:** 10.4102/jphia.v16i1.717

**Published:** 2025-05-14

**Authors:** Genevieve Andoseh, Lionel U. Tiani, Cyriaque A. Ambassa, Diane Kamdem Thiomo, Jean Paul Assam Assam, Cedric F. Tchinda, Leonard N. Numfor, Francine Ntoumi, Véronique Penlap Beng

**Affiliations:** 1 Department of Biochemistry, Faculty of Science, University of Yaoundé I, Yaoundé, Cameroon; 2 Laboratory for Tuberculosis Research and Pharmacology, Biotechnology Center of Nkolbisson (BTC), University of Yaoundé I, Yaoundé, Cameroon; 3 Laboratory for Vector Biology and Control, Biotechnology Center of Nkolbisson (BTC), University of Yaoundé I, Yaoundé, Cameroon; 4 Department of Microbiology, Faculty of Science, University of Yaoundé I, Yaoundé, Cameroon; 5 Center for Research on Medicinal Plants and Traditional Medicine, Institute of Medical Research and Medicinal Plants Studies (IMPM), Ministry of Scientific Research and Innovation, Yaoundé, Cameroon; 6 Congolese Foundation for Medical Research, WHO/AFRO Campus, Brazzaville, Congo; 7 Congolese Foundation for Medical Research, University of Marien Ngouabi, Brazzaville, Congo; 8 Institute for Tropical Medicine, University of Tübingen, Tübingen, Germany

**Keywords:** tuberculosis, COVID-19, infectious agent, COVID-19 knowledge, attitudes, practices, Cameroon

## Abstract

**Background:**

COVID-19 and tuberculosis (TB) were the top two leading causes of death from a single infectious agent in 2022.

**Aim:**

This study aimed at assessing COVID-19 knowledge, attitude, and practices (KAP) and their associated factors among pulmonary TB patients and healthy individuals in Yaoundé, Cameroon.

**Setting:**

The study was conducted at the Jamot Hospital in Yaoundé, a main referral hospital for TB management in Cameroon.

**Methods:**

A cross-sectional design was used to recruit a consecutive sample of TB patients and healthy participants at Jamot Hospital and communities in Yaoundé, Cameroon, from April 2022 to March 2023. Data on socio-demographic characteristics and COVID-19 KAP were collected and analysed using logistic regression with significance considered at *p* < 0.05.

**Results:**

Out of 409 participants, 67.5% had good knowledge, 54% had a favourable attitude, and 22.5% had good practices towards COVID-19. Multivariate analysis identified TB status, age, sex, and marital status as significant factors influencing KAP scores. Good knowledge and good practices were associated with being healthy, young, and single (*p* < 0.05). In addition, the female gender, good knowledge, and favourable attitudes were associated with good practices (*p* < 0.05).

**Conclusion:**

Gaps in COVID-19 KAP among TB patients highlight the need for targeted public health interventions, with a focus on TB patients, males, the elderly, and married individuals for better control.

**Contribution:**

Tuberculosis patients are not adopting positive prevention practices as required, thus increasing their risk of getting COVID-19 and transmitting TB, necessitating urgent action.

## Introduction

Infectious diseases such as tuberculosis (TB) and COVID-19 have had devastating effects on humanity. In fact, TB has been in the history of mankind for over 70 000 years. Nearly 2 billion people are currently infected worldwide, and one-third of the world’s population has latent TB *Bacillus* spp. and is at risk of developing active disease.^1^ Worldwide, an estimated 10.6 million people developed TB in 2022, marking a rise in the TB incidence rate by 3.9% between 2020 and 2022.^2^ This contrasts with COVID-19, caused by the coronavirus severe acute respiratory syndrome coronavirus 2 (SARS-CoV-2), which emerged in December 2019 in Wuhan, China, and became a pandemic on 11 March 2020.^3^ According to the World Health Organization (WHO), the number of infections has surpassed 770m, with approximately 7m deaths reported worldwide, and Africa has confirmed more than 9m cases.^4^ The global response to COVID-19 still remains inadequate and COVID-19 has negatively impacted many areas, including TB care. Estimates have shown that there will be an excess of 6m TB cases by 2025 because of COVID-19.^5^ In fact, data continues to accumulate and become available on SARS-CoV-2, with a lot of common ground with TB. Both TB and COVID-19 primarily affect the respiratory system and can spread through the air and close contact. People having TB and COVID-19 show similar symptoms, such as cough, fever, and difficulty in breathing. Older age, malnutrition, poverty, and certain comorbidities such as diabetes mellitus and chronic obstructive pulmonary disease are risk factors for both diseases. According to a study conducted in Cameroon,^6^ the co-infection of SARS-CoV-2 ribonucleic acid (RNA) with pulmonary TB (PTB) was already found to be 24.3%. Although COVID-19 gained more attention and the pandemic shifted to an endemic situation, there needs to be continuous monitoring of the COVID-19 epidemiological situation and its impact on health systems.^4^ People’s knowledge, attitudes, and practices (KAPs) towards COVID-19 have been used to identify health areas needing particular attention.^7^ Studies have shown that a lack of knowledge about COVID-19 affects their behaviours, directly increasing their risk of infection, while knowledge of the disease and attitudes regarding preventive measures influence the practice of infection prevention.^7^ With an estimated population of over 27.6m in 2022, Cameroon, a country in Central Africa, had progressively adopted WHO strategies according to their context, such as the mandatory use of masks, self-isolation or quarantine, hand washing, and social distancing, while preparing the health systems for specific diagnoses, treatments, and vaccinations.^8,9,10^ According to the WHO, the best weapon to effectively fight COVID-19 remains the COVID-19 vaccine, and the plan was to achieve a vaccination rate of 70% by June 2022. Still, most of these measures have not been sustainably achieved. Vaccination against COVID-19 in Cameroon was launched in March 2021 with the global target of achieving 70% vaccination coverage by the end of the year, and yet only about 5% of the eligible population was vaccinated as of 18 November 2022.^9,10^ This exposes the Cameroonian population to risks and necessitates the examination of KAPs for vulnerable groups. Most studies on COVID-19 KAPs in Cameroon have mainly focused on the general public.^11,12,13,14^ but researchers have not conducted any study among TB patients, who represent a high-risk group. Therefore, firstly this study aimed to assess the level of KAP towards COVID-19 and their associated factors among PTB patients at a main TB referral hospital in Yaoundé, Cameroon, and among healthy individuals living in communities within and outside of Yaoundé, and secondly to get the reasons driving vaccination and COVID-19 testing hesitance to inform policymakers in Cameroon about the best strategies to improve on preventive measures that will go a long way in eradicating COVID-19 and TB, as well as to prepare for any future pandemic.

## Research methods and design

### Study design

This was a cross-sectional survey conducted from April 2022 to March 2023. A structured questionnaire and consent form were given to the respondents.

### Setting

The study took place at the Jamot Hospital in Yaoundé (HJY), which serves as one of the major referral hospitals for TB management in Cameroon. Yaoundé is the capital city of Cameroon, and it is the region where the first COVID-19 case was confirmed on 06 March 2020, before spreading to the other regions.^11^

### Study population and sampling strategy

Study participants included two groups: confirmed PTB patients consulting at HJY and healthy individuals living in Yaoundé and other parts of the country. Eligibility criteria for participation in the survey included being 18 years or older, living in Cameroon, voluntary participation, and the ability to provide informed consent. For the patient group, only those with sputum smear-positive TB and who were willing to partake in the study were enrolled. Patients with extra-PTB and those on anti-TB treatment for more than 2 weeks at the time of recruitment were not included in this study. All participants who wished to withdraw from the study were excluded. Participation in this study was consensual and voluntary, for which all prospective respondents provided informed consent. Participants were recruited using a consecutive sampling technique.

In this study, the sample size was calculated following the formula proposed by Lorentz, stated as Equation 1:

$$N=\left[z^{2}\times p(1-p)\right]/{\text{e}}^{2}$$ [Eqn 1]

where *N* is the sample size, *z* equals 1.96 (value of the 95% confidence interval [CI]), *p* is the standard deviation (prevalence) taken at 50%, and e is the margin of error taken at 5%. Therefore, *N* = [1.96^2^ × 0.5 (1–0.5)]/0.05^2^ = 384.16. However, our population size was 324 TB patients, and we included 85 healthy participants for comparisons in KAP towards COVID-19. This low sample size can be attributed to a lack of willingness to take part in the study because of conflicting requirements needed for another parallel study.

Patients who came to HJY received appropriate consultation and care. After each consultation, the patients were informed about the study, its objectives, and the importance of participating in the survey to achieve these objectives. Once the patients provided informed consent, the investigator asked questions in simple terms and in the patients’ preferred language, allowing them to seek clarification. Participants’ responses were obtained freely and without guidance, allowing for the comprehensive and accurate collection of data. Our healthy participants came from two distinct groups: healthy blood donors from the hospital’s blood bank, who regularly undergo thorough screenings for multiple health issues, and their contacts in the community, ensuring a diverse demographic. They were selected based on the absence of TB symptoms and no prior TB history or exposure, verified through detailed medical histories and physical examinations. Random selection from this eligible pool of healthy blood donors and their contacts minimised selection bias and enhanced the representativeness of our sample. All selected healthy individuals tested negative for human immunodeficiency virus (HIV).

### Data collection

A well-structured interviewer-administered questionnaire, adapted from the WHO survey tool and guidelines for monitoring knowledge, risk perceptions, preventive behaviours, and trust to inform pandemic outbreak response (https://iris.who.int/bitstream/handle/10665/333549/WHO-EURO-2020-696-40431-54222-eng.pdf?sequence=1), was used to collect the data, besides similar published work.^15^ The questionnaire was tailored to the study population and context to make it more user-friendly. The final questionnaire for this study contained 30 questions. The first part gathered seven items on sociodemographic information, including age, gender, profession, level of education, marital status, religion, and place of residence. The second part assessed KAPs related to COVID-19, including vaccine immunisation. The ‘knowledge’ section included five items surveying COVID-19 awareness. The ‘attitudes’ section comprised 11 items, including trust in different sources of information regarding COVID-19, attitudes towards COVID-19 vaccines, and voluntary COVID-19 testing. The ‘practices’ section evaluated seven items, including handwashing, avoiding face-touching, using disinfectants, use of antibiotics for COVID-19, wearing masks, disinfecting surfaces, and vaccination status. Additionally, barriers to some good practices in testing and vaccination were evaluated, with options provided by the research team. Participants responded to a series of yes-or-no and Likert scale questions. Likert scale questions had an agreement scale with three categories (no trust, very little trust, and a great deal of trust), four categories (very difficult, difficult, very easy, and easy), and five categories (strongly agree, agree, neutral, disagree, and strongly disagree). A copy of the study questionnaire is provided as Online Appendix 1.

### Data analysis

Completed questionnaires were entered into CS Pro software and exported to STATA version 14 software for statistical analysis. Dependent variables included participants’ KAP towards COVID-19, while independent variables comprised TB status and sociodemographic factors such as age, gender, educational level, marital status, occupation, religion, and place of residence. Before analysis, we utilised a combination of ‘yes/no’ options and Likert scale options to score participants’ KAP related to COVID-19, and these dependent variables were operationally defined as follows.

#### Knowledge

‘Yes’ answers received a score of 1, while ‘no’ answers were scored as 0. Likert scale responses categorised as ‘very easy’ and ‘easy’ were deemed good knowledge and assigned a score of 1, whereas ‘very difficult’ and ‘difficult’ responses were considered poor knowledge and scored as 0. The total knowledge score ranged from zero to five. Using the 80% score value of 5, participants scoring 4 and above were categorised as having good knowledge, while scores below 4 were considered poor knowledge.

#### Attitude

Similarly, for attitude, Likert scale responses such as ‘very little’ and ‘much trust’ were considered favourable and received a score of 1, while ‘no trust’ responses were considered unfavourable and scored 0. The total attitude score ranged from 0 to 11, with a higher score indicating a more favourable attitude towards COVID-19. A cut-off level of 9–11 (80% score value of 11) was set for a favourable attitude towards COVID-19. A score of 6–8 (50% score value of 11) was set for a neutral attitude, and scores below 6 for an unfavourable attitude.

#### Practice

As for practice, questions in this section had ‘yes/no’ options, with ‘yes’ answers receiving a score of 1 and ‘no’ answers receiving a score of 0. The total practice score ranged from 0 to 7. The overall practice score was classified as good if it was 6 and above (80% score value of 7), as fair if it was between 4 and 5 (50% score value of 7), and poor if it was below 4.

Data analysis involved univariate, bivariate, and multivariate techniques. Univariate analysis included descriptive statistical analysis, while bivariate analysis focused on the association between independent factors and KAP. The strength of association between the independent variables and KAP was determined using odds ratio (OR) with a 95% CI and Cramér’s V. The association was considered weak with a Cramér’s V between 0.1 and 0.2, good when it was between 0.2 and 0.4, and strong when it was above 0.4. A multivariate logistic regression model, including independent variables with a Cramér’s V less than 0.4, was applied. The results were tabulated and presented in a narrative style. A *p*-value < 0.05 was considered statistically significant.

### Ethical considerations

Ethical clearance to conduct this study was obtained from the Centre Regional Ethics Committee for Human Health Research (CRERSH-Ce) (No. 1126/CRERSHC/2020). Before beginning the actual data collection process, hospital authorisation was obtained. Also, written informed consent was obtained from each voluntary participant. Furthermore, the confidentiality and privacy of the respondents were maintained.

## Results

### Sociodemographic characteristics of study participants

In this study, 409 participants accepted and completed the survey, of whom 324 (79.2%) had PTB and were classified as a TB-exposed group, while 85 (20.8%) were TB-free healthy participants and classified as a TB non-exposed group. The majority of participants were young adults aged 18–29 years, predominantly male, employed, and had a secondary level of education. Most were singles, identified as Christians, and lived in Yaoundé. Table 1 details the sociodemographic characteristics of all study participants.

**TABLE 1:** Sociodemographic characteristics of study participants.

Categories	All		TB-exposed		TB non-exposed	
	*n*	%	*n*	%	*n*	%
**Participants**	409	100.0	324	79.2	85	20.8
**Age groups (years)**						
18–29	191	46.7	126	38.9	65	76.5
30–49	155	37.9	140	43.2	15	17.6
≥ 50	63	15.4	58	17.9	5	5.9
**Sex**						
Female	129	31.5	93	28.7	36	42.4
Male	280	68.5	231	71.3	49	57.6
**Employment status**						
Employed	289	70.7	258	79.6	31	36.5
Student	93	22.7	41	12.7	52	61.2
Unemployed or retired	27	6.6	25	7.7	2	2.4
**Educational level**						
No formal or primary education	99	24.2	99	100.0	0	0.0
Secondary	189	46.2	172	53.1	17	20.0
College or university	121	29.6	53	16.4	68	80.0
**Marital status**						
Single	232	56.7	166	51.2	66	77.6
Married or cohabitation	152	37.2	135	41.7	17	20.0
Widowed or divorced	25	6.1	23	7.1	2	2.4
**Religion**						
Christianity	363	88.8	282	87.0	81	95.3
Islam	35	8.6	31	9.6	4	4.7
Others	11	2.7	11	3.4	0	0.0
**Place of residence**						
Yaoundé	334	81.7	257	79.3	77	90.8
Others	75	18.3	67	20.7	8	9.5

TB, tuberculosis.

### Knowledge, attitudes, and practices of COVID-19 in relation to tuberculosis status

#### Knowledge of COVID-19 in relation to tuberculosis status

Out of 409 study participants, 67.5% had good knowledge of COVID-19, while 32.5% had poor knowledge. Notably, TB non-exposed individuals demonstrated better knowledge compared to TB-exposed individuals (Table 2). The majority of participants were well informed about protective measures against COVID-19, the vaccine, and what to do if they suspected infection. However, a significant portion found it challenging to find and assess the reliability of COVID-19 information in the media. Detailed results on knowledge of COVID-19 are presented in Table 2.

**TABLE 2:** COVID-19 knowledge, attitudes, and practices in relation to tuberculosis status.

Categories	Items	Yes or No	Tuberculosis Status				Total	
			Exposed		Non-Exposed			
			*n*	%	*n*	%	*n*	%
Knowledge	Easy to find COVID-19 information	Yes	191	59.0	68	80.0	259	63.3
		No	133	41.0	17	20.0	150	36.7
	Easy to understand COVID-19 action information	Yes	214	66.0	78	91.8	292	71.4
		No	110	34.0	7	8.2	117	28.6
	Easy to judge COVID-19 media reliability	Yes	193	59.6	66	77.6	259	63.3
		No	131	40.4	19	22.4	150	36.7
	COVID-19 self-protection	Yes	319	98.5	84	98.8	403	98.5
		No	5	1.5	1	1.2	6	1.5
	Awareness of COVID-19 vaccines	Yes	299	92.3	85	100.0	384	93.9
		No	25	7.7	0	0.0	25	6.1
	**Overall Knowledge Score**								
	Poor knowledge	-	119	36.7	14	16.5	133	32.5
	Good knowledge	-	205	63.3	71	83.5	276	67.5
Attitude	Trust COVID-19 information from television	Yes	232	71.6	67	78.8	299	73.1
		No	92	28.4	18	21.2	110	26.9
	Trust COVID-19 information from newspapers	Yes	228	70.4	68	80.0	296	72.4
		No	96	29.6	17	20.0	113	27.6
	Trust COVID-19 information from health workers	Yes	240	74.1	69	81.2	309	75.6
		No	84	25.9	16	18.8	100	24.4
	Trust COVID-19 information from social media	Yes	199	61.4	46	54.1	245	59.9
		No	125	38.6	39	45.9	164	40.1
	Trust COVID-19 information from radio	Yes	232	71.6	69	81.2	301	73.6
		No	92	28.4	16	18.8	108	26.4
	Trust COVID-19 information from the Ministry of Health	Yes	235	72.5	67	78.8	302	73.8
		No	89	27.5	18	21.2	107	26.2
	Trust COVID-19 information from celebrities/influencers	Yes	186	57.4	41	48.2	227	55.5
		No	138	42.6	44	51.8	182	44.5
	Trust COVID-19 information from WHO	Yes	236	72.8	69	81.2	305	74.6
		No	88	27.2	16	18.8	104	25.4
	Trust COVID-19 information from the national COVID-19 information website	Yes	212	65.4	66	77.6	278	68.0
		No	112	34.6	19	22.4	131	32.0
	Belief in COVID-19 vaccine effectiveness	Yes	241	74.4	58	68.2	299	73.1
		No	83	25.6	27	31.8	110	26.9
	Willingness to get tested for COVID-19 if exposed	Yes	250	77.2	70	82.4	320	78.2
		No	74	22.8	15	17.6	89	21,8
	**Overall attitude score**								
	Unfavourable attitude	-	87	26.8	16	18.8	103	25.2
	Neutral attitude	-	57	17.6	28	33.0	85	20.8
	Favourable attitude	-	180	55.6	41	48.2	221	54.0
Practice	Frequent hand washing with soap and water for at least 20 s (last 7 days)	Yes	284	87.7	85	100.0	369	90.2
		No	40	12.3	0	0.0	40	9.8
	Avoiding touching eyes, nose, and mouth with unwashed hands (last 7 days)	Yes	153	47.2	65	76.5	218	53.3
		No	171	52.8	20	23.5	191	46.7
	Using disinfectants to clean hands in absence of soap and water (last 7 days)	Yes	152	46.9	59	69.4	211	51.6
		No	172	53.1	26	30.6	198	48.4
	Using antibiotics to prevent or treat COVID-19 (last 7 days)	Yes	63	19.4	4	4.7	67	16.4
		No	261	80.6	81	95.3	342	83.6
	Wearing face coverings in public (last 7 days)	Yes	319	98.5	66	77.6	385	94.1
		No	5	1.5	19	22.4	24	5.9
	Disinfecting surfaces (last 7 days)	Yes	100	30.9	38	44.7	138	33.7
		No	224	69.1	47	55.3	271	66.3
	Vaccinated against COVID-19	Yes	27	8.3	8	9.4	35	8.6
		No	297	91.7	77	90.6	374	91.4
	**Overall practice score**								
	Poor practice	-	96	29.6	6	7.1	102	24.9
	Fair practice	-	153	47.2	62	72.9	215	52.6
	Good practice	-	75	23.2	17	20	92	22.5
	Total	-	324	79.2	85	20.8	409	100.0

WHO, World Health Organization.

#### Attitude towards COVID-19 in relation to tuberculosis status

In this study, most of the participants had a favourable attitude towards COVID-19 (54.0%), while 20.8% had a neutral attitude and 25.2% had unfavourable attitudes (Table 2). Trust in COVID-19 information was mostly from health workers, followed by the WHO, the Ministry of Health, and various media sources (Table 2). The TB-exposed group showed more ‘favourable attitudes’ as compared to the TB non-exposed group. However, TB non-exposed participants had higher trust in information from most sources, except social media, and celebrities, which were more trusted by the TB-exposed group.

Perceptions about the effectiveness of the COVID-19 vaccine and willingness to get tested were generally favourable, with some differences between the TB-exposed and non-exposed groups. Detailed attitudes and perceptions are presented in Table 2.

#### Practices towards COVID-19 in relation to tuberculosis status

In this study, most of the participants demonstrated fair practices towards COVID-19 prevention (52.6%), followed by 24.9% showing poor practices and 22.5% exhibiting good practice. Most respondents adhered to wearing face masks s in public and frequent hand washing. However, practices such as avoiding touching the face with unwashed hands, using disinfectants to clean hands when soap and water were not available, and disinfecting surfaces were less common. A small percentage had taken the COVID-19 vaccine. The TB-exposed group showed slightly better adherence to good practices compared to the non-exposed group. Detailed prevention practices are presented in Table 2.

### Factors associated with knowledge, attitude, and practice of study participants towards COVID-19

This study used logistic regression analysis to investigate significant variables associated with COVID-19 KAP, with the dependent variable being the KAP score towards COVID-19. The variables in Table 1 were included in this analysis, with the principal explanatory variable being TB status (exposed or non-exposed). The OR with a 95% CI was calculated for each significant variable (Table 3).

**TABLE 3:** Factors associated with knowledge of COVID-19 among study participants.

Variables	Knowledge scores				Total (Ligns)		χ^2^ *p*-values (Chi^2^)	COR	95% CI	*p*	AOR	95% CI	*p*
	Poor knowledge		Good knowledge										
	*n*	%	*n*	%	*n*	%							
**Tuberculosis status**													
Exposed	119	89.5	205	74.3	324	100	0.000	1.000	-	-	1.000	-	-
Non-exposed	14	10.5	71	25.7	85	100	-	2.940	1.59–5.45	0.001	2.420	1.29–4.54	0.006
**Age (years)**													
18–29	45	23.6	146	76.4	191	100	0.000*	1.000	-	1.000†	-	-	-
30–49	55	35.5	100	64.5	155	100	-	0.560	0.35–0.90	0.016	0.670	0.41–1.09	0.110
≥ 50	33	52.4	30	47.6	63	100	-	0.280	0.15–0.51	0.000	0.370	0.19–0.70	0.002
**Sex of participants**													
Female	43	33.3	86	66.7	129	100	0.811	1.000	-	-	-	-	-
Male	90	32.1	190	67.9	280	100	-	0.950	0.608–1.476	0.811	NA	-	-
**Employment status**													
Employed	115	39.8	174	60.2	289	100	0.000	1.000	-	-	-	-	-
Student	9	9.7	84	90.3	93	100	-	6.170	2.98–12.76	0.000	NA	-	-
Unemployed or retired	9	33.3	18	66.7	27	100	-	1.320	0.57–3.04	0.512	-	-	-
**Education level**													
No formal or primary	63	63.6	36	36.4	99	100	0.000	1.000	-	-	-	-	-
Secondary	65	34.4	124	65.6	189	100	-	3.340	2.01–5.55	0.000	NA	-	-
College or university	5	4.1	116	95.9	121	100	-	40.600	15.17–108.66	0.000	-	-	-
**Marital status**													
Single	58	25.0	174	75.0	232	100	0.001	1.000	-	1.000	-	-	-
Married or cohabitation	63	41.4	89	58.6	152	100	-	0.470	0.30–0.73	0.001	0.550	0.35–0.87	0.010
Widowed or divorced	12	48.0	13	52.0	25	100	-	0.360	0.16–0.84	0.017	0.460	0.20–1.09	0.079
**Religion**													
Christianity	116	32.0	247	68.0	363	100	0.172	0.800	0.42–1.52	0.496	NA	-	-
Islam or others	17	37.0	29	63.0	46	100	-	1.000	-	-	-	-	-
**Place of residence**													
Yaoundé	100	29.9	234	70.1	334	100	0.019	1.000	-	1.000	-	-	-
Others	33	44.0	42	56.0	75	100	-	1.839	1.101–3.069	0.020	0.670	0.39–1.14	0.140

CI, confidence interval; COR, crude odds ratio; AOR, adjusted odds ratio.

† , TB-unexposed (AOR = 2.3, 95% CI = 1.2–4.4, *p* = 0.01).

### Factors associated with knowledge of COVID-19 among study participants

Knowledge of COVID-19 was significantly associated with all variables except sex and religion of the participants. Two models were specified, with TB status remaining significant in both when adjusted for other variables. The first model included TB status, marital status, and place of residence, and the second model included TB status, age, and place of residence. Per our study, TB non-exposed individuals were significantly twice more likely to have good knowledge related to COVID-19 compared to exposed individuals in both models. In the first model, married participants were significantly less knowledgeable than singles, while in the second model, older age (≥ 50 years) was significantly less knowledgeable compared to those between 18 years and 29 years. Detailed results of the logistic regression analysis are presented in Table 3.

### Factors associated with attitude towards COVID-19 among study participants

Attitude towards COVID-19 was significantly associated with education level, knowledge, and practice. Participants with a university level of education were significantly more likely to have favourable attitudes compared to those without formal or primary education. Participants with poor knowledge were less likely to have favourable attitudes, whereas those with fair and good practices were more likely to have favourable attitudes. The results indicated a weak association between attitude and educational level (Cramér’s V = 0.1652). Table 4 presents the detailed OR and CIs for the significant variables.

**TABLE 4:** Factors associated with attitude towards COVID-19 among study participants.

Variables	Attitude scores						Total (Ligns)		χ^2^ *p*-value (Chi^2^)	COR	95% CI	*p*	AOR (95% CI)
	Unfavourable attitude		Neutral attitude		Favourable attitude								
	*n*	%	*n*	%	*n*	%	*n*	%					
**Tuberculosis status**													
Exposed	87	84.5	57	67.1	180	81.4	324	100	0.228	0.81	0.43–1.52	0.507	NA
Non-exposed	16	15.5	28	32.9	41	18.6	85	100	-	1.00	-	-	-
**Age group**													
18–29	43	22.5	45	23.6	103	53.9	191	100	0.511	1.00	-	-	NA
30–49	40	25.8	30	19.4	85	54.8	155	100	-	0.89	0.53–1.49	0.650	-
≥ 50	20	17.5	10	17.5	33	14.5	63	100	-	0.69	0.36–1.33	0.268	-
**Sex of participants**													
Female	29	22.5	27	20.9	73	56.6	129	100	0.678	1.00	-	-	NA
Male	74	26.4	58	20.7	148	52.9	280	100	-	0.80	0.48–1.33	0.379	-
**Employment status**													
Employed	77	26.6	58	20.1	154	53.3	289	100	0.138	1.00	-	-	NA
Student	17	18.3	25	26.9	51	54.8	93	100	-	1.50	0.81–2.77	0.195	-
Unemployed or retired	9	33.3	2	7.4	16	59.3	27	100	-	0.89	0.38–2.10	0.789	-
**Education level**													
No formal or primary	34	34.3	16	16.2	49	49.5	99	100	0.002	1.00	-	-	NA
Secondary	52	27.5	33	17.5	104	55.0	189	100	-	1.39	0.80–2.40	0.243	-
University	17	14.0	36	29.8	68	56.2	121	100	-	2.78	1.39–5.53	0.004	-
**Marital status**													
Single	54	23.3	54	23.3	124	53.4	232	100	0.400	1.00	-	-	NA
Married or cohabitation	41	27.0	29	19.1	82	53.9	152	100	-	0.87	0.53–1.43	0.583	-
Widowed or divorced	8	32.0	2	8.0	15	60.0	25	100	-	0.82	0.33–2.04	0.664	-
**Religion**													
Christianity	89	24.5	77	21.2	197	54.3	363	100	0.915	1.29	0.64–2.61	0.477	-
Islam or others	14	30.4	8	17.4	24	52.2	46	100	-	1.00	-	-	NA
**Place of residence**													
Yaoundé	79	23.6	72	21.6	183	54.8	334	100	0.299	1.00	-	-	NA
Others	24	32.0	13	17.3	38	50.7	75	100	-	0.68	0.39–1.22	0.195	-
**Knowledge score**													
Poor knowledge	55	41.4	25	18.8	53	39.8	133	100	0.000	0.28	0.17–0.45	0.000	NA
Good knowledge	48	17.4	60	21.7	168	60.9	276	100	-	1.00	-	-	-
**Practice score**													
Poor practice	38	37.2	21	20.6	43	42.2	102	100	0.001	1.00	-	-	NA
Fair practice	53	24.6	47	21.9	115	53.5	215	100	-	1.92	1.11–3.31	0.019	-
Good Practice	12	13.0	17	18.5	63	68.5	92	100	-	4.64	2.18–9.88	0.000	-

CI, confidence interval; COR, crude odds ratio; AOR, adjusted odds ratio.

### Factors associated with prevention practices of COVID-19 among study participants

Factors such as age, sex, employment status, educational level, marital status, knowledge, and attitude scores were all significantly associated with good practice. Younger participants (18–29 years), females, students, those with higher education levels, and those divorced demonstrated more good practices. Two models were specified, with TB status remaining significant in both depending on the other factors associated with it. The first model included TB status, age, and sex and the second included TB status, employment status, sex, and marital status. In the first model, TB status remained significant when adjusted for either age or sex but not both. Ages 30–49 years and > 50 years were significantly less likely to have good practices compared to younger age, that is, 18–29 years. Also, males were less likely to have good practices.

In the second model, TB status remained significant when adjusted for either sex, marital status, or both. However, COVID-19 good practices were significantly thrice higher among TB non-exposed respondents than among TB-exposed respondents. Males and married participants were, respectively, twice less likely and thrice less likely to have good practices compared to their referenced counterparts. Detailed associations and adjusted OR are presented in Table 5.

**TABLE 5:** Factors associated with practice of COVID-19 among study participants.

Variables	Practice scores						Total (Ligns)		χ^2^ *p*-values (Chi^2^)	COR	95% CI	*p*	AOR	95% CI	*p*
	Poor practice		Fair practice		Good practice										
	*n*	%	*n*	%	*n*	%	*n*	%							
**Tuberculosis status**															
Exposed	96	94.1	153	71.2	75	81.5	324	100	0.007	1.000	-	-	1.00	-	-
Non-exposed	6	5.9	62	28.8	17	18.5	85	100	-	3.630	1.36–9.65	0.010	3.35	1.18–9.50	0.023
**Age (years)**															
18–29	31	16.2	106	55.5	54	28.3	191	100	0.000	1.000	-	-	1.00†	-	-
30–49	45	29.0	82	52.9	28	18.1	155	100	-	0.360	0.19–0.68	0.002	0.43	0.22–0.84	0.014
≥ 50	26	41.3	27	42.8	10	15.9	63	100	-	0.220	0.09–0.52	0.001	0.28	0.12–0.68	0.005
**Sex of participants**															
Female	25	19.4	63	48.8	41	31.8	129	100	0.007	1.000	-	-	1.00	-	-
Male	77	27.5	152	54.3	51	18.2	280	100	-	0.400	0.22–0.74	0.004	0.45	0.23–0.86	0.015
**Employment status**															
Employed	90	31.1	146	50.5	53	18.4	289	100	0.000	1.000	-	-	-	-	-
Student	5	5.4	58	62.4	30	32.3	93	100	-	10.190	3.73–27.86	0.000	-	-	-
Unemployed or retired	7	25.9	11	40.7	9	33.3	27	100	-	2.180	0.77–6.20	0.143	-	-	-
**Education level**															
No formal or primary	46	46.5	41	41.4	12	12.1	99	100	0.000	1.000	-	-	NA	-	-
Secondary	51	27.0	101	53.4	37	19.6	189	100	-	2.780	1.30–5.97	0.009	-	-	-
College or university	5	4.1	73	60.3	43	35.5	121	100	-	32.970	10.72–101.35	0.000	-	-	-
**Marital status**															
Single	43	18.5	124	53.4	65	28.0	232	100	0.002	1.000	-	-	1.00	-	-
Married or cohabitation	50	32.9	80	52.6	22	14.5	152	100	-	0.290	0.16–0.55	0.000	0.30	0.16–0.58	0.000
Widowed or divorced	9	36.0	11	44.0	5	20.0	25	100	-	0.370	0.12–1.17	0.091	0.39	0.12–1.28	0.120
**Religion**															
Christianity	90	24.8	193	53.2	80	22.0	363	100	0.897	0.890	0.38–2.09	0.787	NA	-	-
Islam or others	12	26.1	22	47.8	12	26.1	46	100	-	1.000	-	-	-	-	-
**Place of residence**															
Yaoundé	79	23.7	175	52.4	80	24.0	334	100	0.230	1.000	-	-	-	-	-
Others	23	30.7	40	53.3	12	16.0	75	100	-	0.520	0.24–1.11	0.089	NA	-	-
**Knowledge score**															
Poor knowledge	67	50.4	55	41.4	11	8.3	133	100	0.000	0.070	0.03–0.15	0.000	-	-	-
Good knowledge	35	12.7	160	58.0	81	29.3	276	100	-	1.000	-	-	-	-	-
**Attitude score**															
Unfavourable attitude	38	36.9	53	51.5	12	11.7	103	100	0.001	1.000	-	-	-	-	-
Neutral attitude	21	24.7	47	55.3	17	20.0	85	100	-	2.560	1.03–6.38	0.043	-	-	-
Favourable attitude	43	19.5	115	52.0	63	28.5	221	100	-	4.640	2.178–9.881	0.000	-	-	-

CI, confidence interval; COR, crude odds ratio; AOR, adjusted odds ratio.

† , TB non-exposed (AOR = 2.68, 95% CI = 0.97–7.45, *p* = 0.058); male sex (AOR = 0.51, 95% CI = 0.27–0.98, *p* = 0.044).

### Factors driving COVID-19 vaccine hesitancy

**FIGURE 1:**
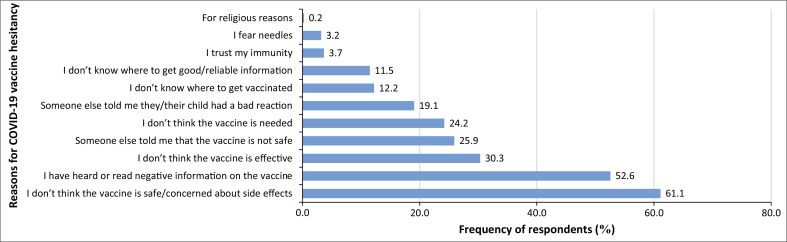
Reasons for COVID-19 vaccine hesitancy and their reported frequencies.

Out of the 409 participants, 35 (8.6%) were vaccinated, while 374 (91.4%) were unvaccinated. Among the TB-exposed, 297 (91.7%) were unvaccinated, while 77 (90.6%) were unvaccinated among the TB non-exposed. The reasons for unwillingness to accept the COVID-19 vaccine were explored (Figure 1). Most participants were hesitant about the vaccination because of concerns related to the safety of the vaccine/side effects (61.1%), hearing or reading negative information on the vaccine (52.6%), vaccine effectiveness (30.3%), being told that the vaccine is not safe (25.9%), and other reasons summarised in Figure 1.

### Factors driving COVID-19 diagnosis hesitancy

**FIGURE 2:**
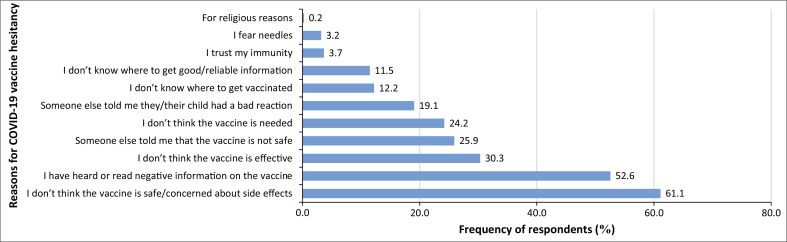
Reasons for hesitancy toward COVID-19 testing and their reported frequencies.

Overall, 320 (78.2%) out of the 409 participants were willing to get tested if they were at risk and given the opportunity. Nevertheless, 89 (21.8%) were unwilling to do a COVID-19 test, among which 22.8% came from the TB-exposed group and 17.6 % came from the TB non-exposed group. The reasons for COVID-19 diagnosis hesitancy were also explored, as illustrated in Figure 2. The main reasons for refusal to do a COVID-19 test included not thinking the tests are reliable (9.5%), not believing COVID-19 exists (8.3%), and fearing being infected at the test site (8.1%). Other reasons included the fear of the test being painful (2.7%), not trusting authorities with one’s personal data (2.4%), not being able to isolate oneself if the test is positive (1.7%), fear of being poorly treated if the test is positive (1.7%), not knowing where to go and do the test (0.7%), and the feeling of an inability to do anything even if the test is positive (0.2%).

## Discussion

The interplay between TB and COVID-19, given their overlapping modes of transmission, exacerbates the risk each poses to the other.^3^ This pioneering epidemiological survey in Cameroon is the first to delve into the KAP of TB patients regarding COVID-19 and provides vital insights into designing targeted public health interventions aimed at mitigating the dual threat of these infectious diseases. We assessed the KAPs related to COVID-19 and its associated factors among PTB patients at a TB referral hospital in Yaoundé, Cameroon, and compared these findings with healthy individuals within and outside Yaoundé. Additionally, we sought to identify the factors driving COVID-19 vaccine and testing hesitancy, which will be valuable for local authorities to address. The results illuminate significant gaps and opportunities for public health education and intervention.

### Knowledge of COVID-19

Our study reports a high level of awareness among the 409 study participants (67.5%), with healthy participants (83.5%) having better knowledge than TB patients (63.3%). These results are comparable to similar studies in Egypt and Nigeria (61.6%),^16^ in Northern Ethiopia (74.2%),^17^ in Cameroon (78.6% and 80%),^12,13^ and in Malaysia (80.5%).^18^ In contrast, the knowledge level found in this study was lower than previous KAP studies in Cameroon (84.2%)^11^ and China (90%).^7^ Differences in the assessment tools used, the criteria for classifying knowledge levels, education levels and demographics, study settings, and the timing of data collection likely influenced these variations.

### Attitudes towards COVID-19

In our study, we assessed participants’ attitudes towards COVID-19 prevention measures, revealing varying levels of trust in information sources. A majority (54.0%) held favourable attitudes, while 20.8% were neutral, and 25.2% had unfavourable attitudes. These findings align with other studies showing similar rates of favourable attitudes in Yemen,^19^ Cameroon,^11,12^ Ethiopia,^3,17^ Kenya,^20^ and among Egyptians and Nigerians.^16^ However, the rate of those with favourable attitudes was lower compared to those reported in Malaysia (83.1%)^18^ and higher than a study conducted in Cameroon (28%).^13^ The variation in results may be attributed to differences in study area and population, cut-off values for classifying attitudes, knowledge levels, and sociodemographic characteristics of the participants. Understanding the levels of trust and protective behaviours in various sociocultural settings is essential. Participants with favourable attitudes trusted health workers (75.6%), the WHO (74.6%), and the Ministry of Health (73.8%) the most. Conversely, social media (59.9%) and celebrities/influencers (55.5%) were least trusted. This trend is consistent with findings from other studies, emphasising the crucial role of healthcare professionals and government communications in disseminating reliable information about pandemic risks and the benefits of adhering to protective measures.^21,22^ Tuberculosis patients showed more favourable attitudes (55.6%) compared to healthy participants (48.2%), partly because of stronger trust in social media and celebrities. Contrarily, TB patients were less inclined to undergo voluntary COVID-19 testing (77.2%) compared to healthy participants (82.4%). Improving testing willingness, especially within the TB-affected population, is essential for better public health outcomes.

### Practices towards COVID-19 prevention measures

Our study found that only 22.5% of the participants demonstrated good practices in general. Similar low levels of good practice were observed in studies conducted in Cameroon (23.1%) and Northern Ethiopia (39.2%).^12,17^ This contrasts with higher levels of good practices reported in other studies in Cameroon (51.6% and 60.8%),^11,13^ Northern Sri Lanka (60.8%),^23^ and among Egyptians and Nigerians (96%).^16^ Factors such as study period, socioeconomic status, differing criteria used to classify good or poor practice, and access to preventive measures likely influenced these differences. Regarding good practices in our study, the majority of respondents wore face masks in public (94.1%) and frequently washed their hands with soap and water for at least 20 s (90.2%). These findings are consistent with other studies, showing a high proportion of participants adhering to the use of masks^7,11,20,23,24^ and hand washing with soap.^11,18,20,23,24^ Conversely, in other studies, the percentage of individuals who wore a mask and practised hand washing was lower, as seen in Yemen (25% and 23.25%, respectively),^19^ and 26.5% regularly wore a mask in Northern Ethiopia.^17^ The high adherence to these two prevention practices in our study can be attributed to strict government measures.^11^

Wearing face masks is crucial for preventing TB and COVID-19 transmission. In our study, 98.5% of the TB patients wore face masks, compared to 77.6% of the healthy individuals. Some TB patients who did not initially wear masks were provided with them during the study, but they may have unknowingly transmitted TB in their environment before seeking medical care. Historically, wearing a mask to prevent TB transmission has faced scepticism and low acceptance because of stigma and perceived loss of freedom.^5^ Despite good knowledge and favourable attitudes towards COVID-19, adherence to preventive practices was low. This could be because of the misconception that COVID-19 is no longer a threat and therefore preventive measures are not adhered to. It is important to address and dispel these misconceptions to improve adherence to preventive measures.

### Factors associated with COVID-19 knowledge, attitudes, and practices among study participants

Multivariate analysis identified that TB status, age, and marital status significantly impacted knowledge. Healthy individuals were twice as likely to have good knowledge compared to TB patients. This difference could partly be because of the higher educational level among the healthy population in comparison to the TB group. It also indicates substantial knowledge gaps among TB patients, necessitating targeted educational efforts to improve COVID-19 knowledge. Younger participants (18–29 years) had better knowledge than those aged 30–49 years and 50 years and above, with the disparity being significant only for those aged 50 years and above. Studies indicate that individuals aged 50 years and above generally have poorer knowledge compared to younger people,^3,16,23^ possibly because of age-related declines in hearing and vision, which make accessing information harder.^3^ Furthermore, younger individuals, particularly students, have more opportunities to stay informed. However, some studies contradict this, showing older age associated with better knowledge.^17,18^ These inconsistencies could be because of variations in the study population.

Married individuals were found to significantly have lower knowledge levels compared to singles. This finding aligns with previous studies conducted in Kenya and Ethiopia, which found that singles exhibited greater knowledge compared to married individuals.^20,25^ One possible explanation for this could be that singles have fewer social and family responsibilities, enhancing their knowledge. However, this contrasts with another study that found higher COVID-19 knowledge among married individuals.^19^

Furthermore, the analysis showed that TB status, sex, marital status, and age, significantly impacted COVID-19 practice scores. Healthy respondents were observed to be three times more likely to exhibit good practices for COVID-19 compared to TB respondents. This sheds light on the need to promptly address risky behaviours among TB patients. Males were two times and three times less likely to exhibit good practices in comparison to their counterparts. This finding supports existing studies associating higher practice scores with females,^7,18,24^ possibly because of their caregiving roles and concern for family health.^24,26^ Men, being a documented risk factor for both COVID-19 and TB, are more prone to risky behaviours and severe outcomes, necessitating gender-specific strategies in TB and COVID-19 control programmes.^2,7,27^ Single individuals exhibited better adherence than their married counterparts, which aligns with findings from another research.^24^ This could be attributed to the fact that the majority were single participants who possessed good knowledge about COVID-19. Participants aged 30–49 years and ≥ 50 years showed lower adherence to good practices compared to those aged 18–29 years. Older individuals, being more vulnerable because of decreased immune efficiency, require closer monitoring.

Moreover, this study also revealed critical connections between good knowledge, favourable attitudes, and good practices. Individuals with favourable attitudes were 4.6 times more likely to exhibit good preventive practices. This trend is consistent with other studies, indicating that favourable attitudes towards COVID-19 measures enhance adherence.^7,17^

The survey revealed a significant gap between the positive perception of the COVID-19 vaccine’s effectiveness (73.1%) and the actual vaccination rate (8.6%), which is below the national target. Similarly, low vaccination rates (10% and 39.8%) were observed in other studies in Cameroon.^8,10^ A notable 91.4% of participants remain unvaccinated, especially TB patients (91.7%). This indicates an urgent need for increased vaccination efforts, especially in both urban and rural areas of Cameroon.^9^ Vaccine hesitancy is prevalent because of safety concerns (61.1%), exposure to negative information (52.6%), and doubts about effectiveness (30.3%). These findings, consistent with other studies,^9,10,15^ highlight the disinterest of the population towards vaccination and reliance on traditional medicinal practices.^9,10^ To confront this challenge, it is imperative for the Ministry of Health to ramp up efforts to educate the public about vaccination, dispel misinformation, and ensure comprehensive COVID-19 vaccination coverage across the country.

The willingness of citizens to undergo testing significantly impacts the effectiveness of mass testing, making voluntary random testing a highly recommended approach.^28^ This study found that 78.2% of the participants were willing to undergo COVID-19 testing if at risk. Comparable figures from other studies reported percentages of 72.0% in Cameroon and 82.5% in Kenya in favour of voluntary testing.^11,20^ However, 21.8% were unwilling, with higher reluctance among TB patients. Reasons included test unreliability (9.5%), disbelief in COVID-19 (8.3%), and fear of infection at test sites (8.1%). Existing evidence suggests that individuals with a preference for understanding their health status are more inclined to seek voluntary testing, with their decisions influenced by various factors such as pre-existing health conditions, previous healthcare experiences, and the nature and frequency of communication.^28^ A study in Cameroon revealed a relatively high proportion (7.63%) of the population expressing disbelief in the existence of COVID-19,^9^ which may be attributed to the denial phenomenon as an unconscious reflex.^8^

Furthermore, a qualitative study on COVID-19 testing in Bhutan revealed how unpleasant experiences with nasopharyngeal swab testing instilled fear and reluctance in others to get tested. Moreover, the study unveiled a lack of knowledge about how or where to access a test, indicating that distrust, fear, and stigma associated with COVID-19 and public health responses present significant challenges in our settings.^29^

All these findings underscore the urgent need for continuous education and sensitisation campaigns in the population aimed at fostering favourable attitudes and preventive practices in relation to COVID-19.

### Strengths and limitations

#### Strengths

This study conducted in Yaoundé represents the first of its kind to assess the KAPs concerning COVID-19 among TB patients and a healthy control group. Carried out at the HJY, the primary TB referral hospital in the country, the study presents substantiated evidence and identifies prevailing gaps that can effectively guide policymakers in the development of robust public health information dissemination strategies and immediate public health preventive measures.

#### Limitations

While our study on TB patients’ KAPs concerning COVID-19 in Yaoundé, Cameroon, has certain limitations, it presents opportunities for constructive action. The over-representation of respondents from Yaoundé, males, singles, and individuals with at least a secondary education necessitates further investigation into the views and behaviours of uneducated and rural populations in Cameroon. Furthermore, the absence of prior validation of the questionnaire for assessing KAPs related to COVID-19 in this specific context, coupled with the potential inadequacy of the sample size in determining the statistical significance of certain variables, poses limitations to the study’s robustness. However, this limitation opens the door for the development of a tailored questionnaire that aligns with the unique characteristics of the Cameroonian population and serves as a call for future studies to ensure robust sample sizes that can yield more conclusive results. Additionally, as a cross-sectional study, our findings establish associations rather than temporal relationships. Despite these limitations, our findings still offer a basis for promoting health-conscious behaviours and improving patient care through educational and preventive measures at the national level, with potential applications in other African countries grappling with high rates of TB. Nevertheless, further studies are warranted to investigate knowledge gaps and practices among TB patients across various health centres in the country, furthering our potential to positively impact public health initiatives.

## Conclusion

This study comprehensively assessed KAP levels related to COVID-19 among TB patients and healthy individuals. Both groups exhibited good knowledge and attitudes, but their practices towards COVID-19 were lacking. Notably, the healthy individuals displayed superior KAP levels compared to the TB participants. It is imperative for the government to bolster the capabilities of healthcare professionals in improving communication strategies that enhance good practices related to COVID-19, particularly within the TB-affected population. Furthermore, there should be intensified efforts to disseminate health education, particularly targeting males, the elderly, and those who are married. Recognising the unique factors observed in the different study groups is pivotal for effectively combating TB and COVID-19 and preventing future infections.
